# Evaluation of phytochemicals and antioxidant potential of a new polyherbal formulation TC-16: additive, synergistic or antagonistic?

**DOI:** 10.1186/s12906-023-03921-0

**Published:** 2023-03-28

**Authors:** Vi Lien Yap, Lee Fang Tan, Mogana Rajagopal, Christophe Wiart, Malarvili Selvaraja, Mun Yee Leong, Puay Luan Tan

**Affiliations:** 1 Faculty of Pharmaceutical Sciences, UCSI University, UCSI Heights 1, Jalan Puncak Menara Gading, Taman Connaught, Cheras, Kuala Lumpur, 56000 Malaysia; 2 Institute for Tropical Biology & Conservation, Universiti Malaysia Sabah, Kota Kinabalu, 88400 Malaysia

**Keywords:** Polyherbal, Turmeric, Ginger, Black pepper, Calamansi, Tualang honey, Phytochemicals, Antioxidant activity, Interaction

## Abstract

**Background:**

Scientific literature has demonstrated the association of free radicals in the aetiology of various chronic diseases. Hence, the identification of potent antioxidants remains a useful task. The combination of multiple herbs in polyherbal formulations (PHF) is often associated with greater therapeutic efficacy due to synergistic interactions. However, antagonism can occur in natural product mixtures and the resultant antioxidant potential might not always be the additive value of the antioxidant properties of each component. In this study, we aimed to evaluate the phytochemicals, antioxidative potential and interaction among the herbs in TC-16, a new PHF comprising *Curcuma longa* L., *Zingiber officinale* var. Bentong*, Piper nigrum* L., *Citrofortunella microcarpa* (Bunge) Wijnands and *Apis dorsata* honey.

**Methods:**

TC-16 was screened for phytochemicals. Phenolic and flavonoid contents of TC-16 and its individual ingredients were determined, followed by assessment of antioxidant properties using in vitro assays including 2,2’-azino-bis(3-ethylbenzothiazoline-6-sulfonate) (ABTS), 2,2-diphenyl-1-picrylhydrazyl (DPPH), ferric reducing antioxidant power (FRAP), oxygen radical absorbance capacity (ORAC) and β-carotene bleaching (BCB) assays. Interactions among the herbs were also investigated by calculating the difference in antioxidant activity and combination index.

**Results:**

Alkaloids, flavonoids, terpenoids, saponins and glycosides were present in TC-16. TC-16 possessed the highest phenolic (46.14 ± 1.40 mg GAE/g) and flavonoid (132.69 ± 1.43 mg CE/g) contents following *C. longa*. Synergistic antioxidant activity among the herbs was evident in ORAC and BCB assays which uses mainly hydrogen atom transfer-based antioxidant mechanisms.

**Conclusions:**

TC-16 demonstrated roles in combating free radicals. In a PHF, synergistic interaction among the herbs is observed in some but not all mechanisms. Mechanisms showing synergistic interactions should be highlighted to maximise the beneficial property of the PHF.

## Background

Free radicals play vital roles in the aetiology of numerous diseases including atherosclerosis, cancer, cardiovascular diseases, diabetes, inflammation and neurological disorders due to their ability to damage nucleic acids, lipids, proteins, polyunsaturated fatty acids, and carbohydrates [1]. Studies have reported a positive relationship between the consumption of antioxidants and the reduction of risk of developing chronic and ageing-related diseases [2]. The potential toxicity of synthetic antioxidants, inter alia, butylhydroxyanisole (BHA) and butylhydroxytoluene (BHT) has caused stricter regulations to be imposed on their application and there is a growing interest to replace them with antioxidants naturally present in plants [3].

Plants are valuable sources of bioactive compounds with prominent pharmacological effects, low toxicity, and minimal side effects. Numerous plant crude extracts and pure compounds have been reported to demonstrate antioxidant activities, among which vitamin E, vitamin C, and polyphenols/flavonoids are examples of well-known natural antioxidants [4]. Combinations of herbs have been employed in traditional Ayurvedic, Chinese, Unani, and Peruvian practices for millennia for the management of numerous ailments. The concept of polyherbalism is highlighted in Ayurveda, in which a combination of several herbs in a certain ratio will result in a better therapeutic effect and reduced toxicity [5]. However, in mixtures of antioxidants, subject to the reaction environment and the presence of other ingredients in the mixture, the resultant antioxidant capacity might not be the additive value of the antioxidant properties of each individual ingredient [1].

In light of the above, this research was conducted to evaluate the antioxidant potential of a new polyherbal formulation known as TC-16 consisting of *Curcuma longa* L., *Zingiber officinale* var. Bentong*, Piper nigrum* L., *Citrofortunella microcarpa* (Bunge) Wijnands and honey from *Apis dorsata*, formulated with the selection of its components based upon their reported antioxidant activity in the past studies [6–10], to investigate its antioxidant mechanisms as well as to assess the relationship among the herbs of the formulation in term of antioxidant activity. The authors hypothesized that the polyherbal TC-16 will exhibit superior antioxidant activities than its individual ingredients.

## Materials and methods

### Chemicals

Trolox (6-hydroxy-2,5,7,8-tetramethylchroman-2-carboxylic acid), 2,2'-azino-bis(3-ethyl benzothiazoline-6-sulfonic acid) (ABTS) diammonium salt, 2,4,6-tris(2-pyridyl)-s-triazine (TPTZ), gallic acid, aluminium chloride (AlCl_3_), β-carotene and fluorescein sodium salt were procured from Sigma-Aldrich. Magnesium, mercury (II) chloride, 2,2-diphenyl-1-picrylhydrazyl (DPPH), sodium acetate trihydrate, Folin-Ciocalteu phenol reagent, sodium carbonate (Na_2_CO_3_), sodium hydroxide (NaOH) and sodium acetate trihydrate were acquired from Merck. ( +)-Catechin hydrate, Tween 20, linoleic acid, 2,2’-azobis (2- methylpropionamidine) dihydrochloride (AAPH) and di-potassium hydrogen phosphate anhydrous were obtained from ChemSoln. Iron (III) chloride-6-hydrate and sodium nitrite were acquired from Bendosen. Ascorbic acid and gelatine were purchased from R&M Chemicals, potassium dihydrogen phosphate and potassium persulphate were from John Kollin Corporation. Iron (II) sulfate, iodine and ammonia solution were obtained from Fisher Scientific and glacial acetic acid was obtained from Chemiz. Hydrochloric acid (HCl) and sulfuric acid (H_2_SO_4_) were from Univar. Potassium iodide was purchased from Systerm.

### Sample

The formulation TC-16 was prepared by mixing a specific proportion of aqueous extracts of *C. longa*, *Z. officinale* var. Bentong*, P. nigrum* and *C. microcarpa* along with honey from *A. dorsata*. The ratios of each component of TC-16 are shown in Table 1.

**Table 1:** Composition of polyherbal TC-16

Ingredients	Part used	Parts by weight (%, w/w)
*C. longa*	Rhizomes	1 (22.22%)
*Z. officinale*	Rhizomes	1 (22.22%)
*P. nigrum*	Fruits	1 (22.22%)
*C. microcarpa*	Fruits	0.5 (11.11%)
*A. dorsata* honey	-	1 (22.22%)

### Preliminary phytochemical screening

Qualitative phytochemical screening of the herbs was performed as follows [11, 12]:

####  Alkaloids

5 mL of 2 N HCl was added to 10 mL of TC-16 in methanol (20 mg/mL). The mixture was left cooled and filtered after being subjected to heating in a boiling water bath. The filtrate was split into two parts, with each part being tested with a few drops of Mayer’s reagent and Wagner’s reagent respectively.

Mayer’s reagent: A mixture consisting of 60 mL mercuric (II) chloride in water (2.27%w/v) and 10 mL potassium iodide in water (50%w/v) was prepared and diluted to 100 mL. The presence of a white to yellowish precipitate indicates that alkaloids are present.

Wagner reagent: 1.27 g iodine and 2 g potassium iodide were dissolved in 20 mL of water, and this was diluted to 100 mL. Alkaloids were indicated by the presence of brown precipitate.

####   Flavonoids

2 mL of TC-16 in ethanol (2%w/v) was prepared and filtered. A few drops of concentrated HCl and 0.5 g of magnesium ribbon were added to the filtrate. The presence of pink or magenta red colour reveals the presence of flavonoids.

####   Terpenoids

100 mg TC-16 was shaken with 2 mL of chloroform. 2 mL of concentrated H_2_SO_4_ was introduced along the side of the test tube. The reddish-brown colour at the interface shows that terpenoids are present.

####   Tannins

6 mL of TC-16 in hot distilled water (0.17%w/v) was prepared and filtered. The filtrate was portioned into three parts. 0.9% sodium chloride (NaCl), 0.9% NaCl and 1% gelatine solution, and ferric chloride were added to each part respectively. The presence of precipitate in the second part and the presence of blue, blue-black or greenish colour reveal the presence of tannins.

####   Saponins

In 10 mL of distilled water, 0.5 g of TC-16 was added and shaken. The presence of frothing that lasts on warming in a water bath for 5 min indicates that saponins are present.

####  Glycosides

Borntrager’s test: 1 mL of 5% H_2_SO_4_ was added to 1 mL of TC-16 and boiled in a water bath. After filtering, the filtrate was mixed with 2 mL of chloroform and left standing for 5 min. The bottom layer of chloroform was mixed with 50% of its volume of dilute ammonia. The presence of anthraquinone glycosides is shown by the formation of the rose pink to red colour of the ammoniacal layer.

Keller-Killiani test: 2 mL of glacial acetic acid, a few drops of ferric chloride and 1 mL of sulfuric acid along the side of the test tube were added in sequence to 5 mL of 10%w/v TC-16 in water. The presence of a brown ring at the interface shows the presence of cardiac glycosides.

### Total phenolic and flavonoid contents

####  Total phenolic content (TPC) 

The total phenolic content in the samples was analysed by Folin-Ciocalteu (FC) reagent [13]. Gallic acid solutions of 50–250 μg/mL were prepared for constructing the calibration curve. 5 mL of FC reagent (diluted tenfold from 2 M) was added to 1 mL of the solution and was left for 5 min. Subsequently, 5 mL of sodium carbonate solution (75 g/L) was added to the mixture. The absorbance was read spectrophotometrically after 0.5 h at 20 °C at 765 nm. 1 mL of 1 mg/mL sample was used in replacement of gallic acid, and the absorbance was determined after 1 h. The phenolic contents were expressed as mg gallic acid equivalent (GAE) per gram of herbal extract.

####   Total flavonoid content (TFC)

The total flavonoid content of the samples was analysed using aluminium chloride colorimetric assay [6]. A range of catechin solutions was prepared (6.25–200 μg/mL) to obtain the calibration curve. In a flask containing 4 mL of distilled water, 1 mL of catechin solution followed by 0.3 mL of 5% sodium nitrite were added and mixed. After 5 min, 0.3 mL of 10% aluminium chloride was added. 6 min later, 2 mL of 1 M NaOH solution was added followed by the immediate addition of 2.4 mL of distilled water, totalling a final volume of 10 mL. The absorbance of the flavonoid-aluminium complex was obtained at 510 nm. 1 mg/mL of samples was used to determine the flavonoid contents with the results expressed as mg catechin equivalent (CE) per gram of herbal extract.

### Antioxidant capacity tests

####  2,2’-azino-bis(3-ethylbenzothiazoline-6-sulfonate) (ABTS) assay

ABTS assay was carried out as described by Mogana et al*.* [14]. Aliquots of samples in different concentrations were added to a 96-well microtiter plate in triplicate. Trolox and ascorbic acid in different concentrations were employed as positive controls. ABTS^•+^ working solution was obtained by mixing an equal volume of ABTS solution (7 mM) and potassium persulfate solution (2.4 mM). The working solution was protected from light and stored for 12–16 h to stabilize it before use and was stable for not more than 3 days when kept in the dark. The ABTS^•+^  working solution was diluted to the absorbance of 0.70 ± 0.01 at 734 nm at 37 °C with ethanol just before the assay. The antioxidant activity of the samples was determined by adding 100 μL of ABTS ^•+^ solution to 100 μL of the samples. The solution was kept at 37 °C for 7 min before obtaining the percentage decolourisation spectrophotometrically at 734 nm. The ABTS radical scavenging capacity (%) was calculated using the equation:

$$\frac{\mathrm{Abs}\;\mathrm{control}-\mathrm{Abs}\;\mathrm{sample}}{\mathrm{Abs}\;\mathrm{control}}\mathrm x\;100$$

where: Abs control—the absorbance of ABTS radical with ethanol; Abs sample—absorbance of ABTS radical with herbs/standard.

####  2,2-diphenyl-1-picrylhydrazyl (DPPH) assay

DPPH assay was conducted according to Juan-Badaturuge et al*.* [15] to evaluate the antioxidant potential of the herbs. Aliquots of TC-16 and the individual herbs were added to a 96-well microtiter plate in triplicate. 0.1 mM DPPH solution was added to the wells containing the test samples while methanol was added instead of DPPH as the sample control in the remaining wells. The plate was shaken for 120 s and was incubated for 30 min protected from light. The absorbance was read at 550 nm. A graph of percentage radical scavenging activity against concentration was plotted to obtain the EC_50_ values. The percentage of DPPH radical scavenging capacity (%) was calculated using the same equation as described in the ABTS assay.

####   Ferric reducing antioxidant power (FRAP) assay

FRAP assay was carried out according to Mogana et al. [16] with slight modifications. 6.25 μg/mL of samples were plated out in triplicate in a 96-well microtiter plate. FRAP working solution was prepared just before the assay by mixing acetate buffer (300 mM, pH 3.6), TPTZ (10 mM), and FeCl_3_·6H_2_O (20 mM) in the ratio of 10: 1: 1. 180 μL of the FRAP reagent was mixed with 20 μL of the test sample. After 30 min, the absorbance was measured at 593 nm. A series of Fe (II) concentrations (3.125—100 μM) were used as a standard for the calibration curve. The antioxidant activity was expressed as μM FeSO_4_/μg sample. The colour absorbance of samples was corrected using sample wells without FRAP reagent.

####  Oxygen radical absorbance capacity (ORAC) assay

A stock solution of sodium fluorescein (4.19 × 10^−3^ mM) was prepared using 75 mM potassium phosphate buffer (pH 7.4) and it was stored at 4 °C. The working solution (8 × 10^−5^ mM) was prepared freshly by diluting the stock solution in the buffer. The stock solution of Trolox standard (0.02 M) was prepared and further diluted with buffer to give working solutions of 100, 50, 25, 12.5 and 6.25 μM. Lastly, 0.2 g of AAPH was dissolved in 5.0 mL of buffer.

The ORAC assay was conducted according to Huang et al. [17] with slight modification. 25 μL each of Trolox standards, samples and potassium phosphate buffer was added into a 96-well microplate. 150 μL of sodium fluorescein working solution was then added to the wells. The microplate was sealed followed by incubation for 20 min at 37 °C in the microplate incubator with shaking. After incubation, 20 μL of AAPH solution was added lastly into the well. The fluorescence intensity was measured every 88 s for 88 min by BMG Labtech FLUOstar Omega multimode microplate reader where excitation and emission wavelengths were 485 and 520 nm, respectively. The final fluorescence measurements were expressed relative to the initial readings. The area under the curve (AUC) was calculated and the net AUC was obtained by subtracting the AUC for the blank. Linear regression analyses were performed to obtain the slopes of the regression equations. The ORAC value for each sample was calculated by dividing the slope of the sample by the slope of Trolox and expressed as µmol Trolox equivalent (TE) per 1 g sample.

####  β-carotene bleaching (BCB) assay

β-carotene bleaching assay was conducted according to the method described by Mogana et al. [16]. Aliquots of TC-16 and the individual herbs (20 μL) were added to a 96-well microtiter plate in triplicate at different concentrations. 1 mL of β-carotene solution in chloroform (0.2 mg/mL) was added into a round bottom flask containing 40 μL of linoleic acid and 500 μL of Tween 20. Chloroform was removed using a rotary vacuum evaporator at 45 °C followed by the addition of 100 mL of deionised water with vigorous agitation. 80 μL of the emulsion was added to the test samples in 96-well microtiter plate. The absorbance was measured at 470 nm immediately against a blank (emulsion without β-carotene) after 3 h of incubation at 50 °C. The antioxidant activity (%) of the test samples was evaluated in terms of β-carotene bleaching using the formula:

$$(1-\frac{{\mathrm{A}}_{0}-{\mathrm{A}}_{\mathrm{t}}}{{\mathrm{A}}_{0}\mathrm{^{\prime}}-{\mathrm{A}}_{\mathrm{t}}\mathrm{^{\prime}}})\times 100$$

where: A_0_ and A_0_′ are absorbances measured at zero time of incubation for the test sample and control, respectively; A_t_ and A_t_′ are the absorbances of the test sample and control, respectively after 3 h of incubation. Sample well without β-carotene was used as sample blank control to correct colour absorbance of samples.

### Statistical analysis

Data were presented as mean ± SD and were statistically analysed using GraphPad Prism version 8.4.0 software. One-way ANOVA and Tukey’s multiple comparison tests were applied to find out the differences between the means. The significance was established at p < 0.05. The correlation analysis results for the different assays were expressed as Pearson correlation coefficients using SPSS Version 26.0 (SPSS, Chicago, IL, USA).

Interaction between the individual ingredients as determined in FRAP and ORAC assay were calculated using the following equation [18]:

$$\mathrm{Difference}\;(\%)=\frac{\mathrm{Combination}\;\mathrm{abcde}\;\mathrm x\;100}{\mathrm a+\mathrm b+\mathrm c+\mathrm d+\mathrm e}-100$$

where: combination abcde is the result obtained for TC-16, while each a/b/c/d/e values are the value measured individually for each compound.

The interaction quantification for ABTS, DPPH and BCB assay was achieved using a combination index (CI) [19]:

$$\mathrm{CI }= \frac{{\mathrm{IC}}_{50}\mathbf{a}\mathbf{b}\mathbf{c}\mathbf{d}\mathbf{e}/\mathrm{x}}{{\mathrm{IC}}_{50}\mathbf{a}}+\frac{{\mathrm{IC}}_{50}\mathbf{a}\mathbf{b}\mathbf{c}\mathbf{d}\mathbf{e}/\mathrm{x}}{{\mathrm{IC}}_{50}\mathbf{b}}+\frac{{\mathrm{IC}}_{50}\mathbf{a}\mathbf{b}\mathbf{c}\mathbf{d}\mathbf{e}/\mathrm{x}}{{\mathrm{IC}}_{50}\mathbf{c}}+\frac{{\mathrm{IC}}_{50}\mathbf{a}\mathbf{b}\mathbf{c}\mathbf{d}\mathbf{e}/\mathrm{x}}{{\mathrm{IC}}_{50}\mathbf{d}}+\frac{{\mathrm{IC}}_{50}\mathbf{a}\mathbf{b}\mathbf{c}\mathbf{d}\mathbf{e}/\mathrm{x}}{{\mathrm{IC}}_{50}\mathbf{e}}$$

where: IC_50_ **a**/**b**/**c**/**d**/**e** are the values obtained for the individual herbs, while IC_50_ **abcde**/x is the concentration of the individual herbs in the polyherbal that causes 50% inhibition.

## Results

### Preliminary phytochemical screening

Phytochemical screening tests on TC-16 (Table 2) showed the presence of secondary metabolites including alkaloids, flavonoids, terpenoids, saponins and both cardiac and anthraquinone glycosides.

**Table 2:** Qualitative phytochemical screening of polyherbal TC-16

Test	Result
Alkaloids	
(a) Mayer’s test	+
(b) Wagner’s test	+
Flavonoids	
(a) Shinoda test	+
Terpenoids	
(a) Salkowski test	+
Tannins	-
Saponins	+ (0.5 cm foam)
Glycosides	
(a) Borntrager’s test	+
(b) Keller-Killiani test	+

+  = presence, −  = absence

### Determination of phenolic and flavonoid contents and antioxidant capacity tests

**Fig. 1:**
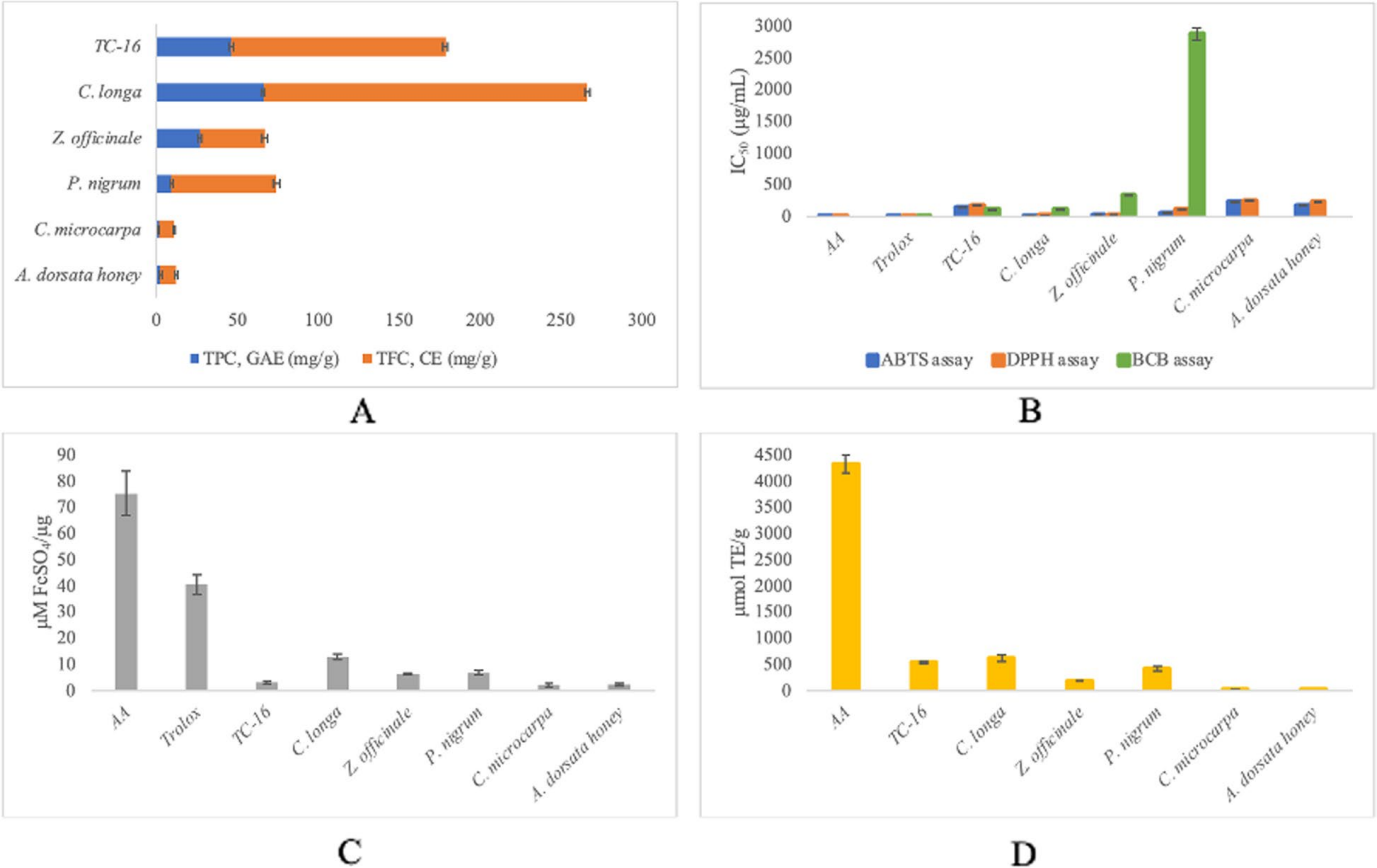
Antioxidant capacity of the individual herbs and TC-16. Data are presented as mean ± SD. **A** TPC (*n* = 9) and TFC (*n* = 9), **B** ABTS, DPPH and BCB assays (*n* = 9), **C** FRAP assay (*n* = 9), **D** ORAC assay (*n* = 6)

The total phenolic and flavonoid contents of the individual herbs and TC-16, as well as the results for in vitro antioxidant assays, were shown in Fig. 1.

### Correlation and interaction analysis

Pearson correlation was conducted (Table 3) to correlate the results obtained from the different assays.

**Table 3:** Pearson’s correlation coefficients of total phenolic and flavonoid contents as well as antioxidant assays

	**TPC**	**TFC**	**ABTS assay**	**DPPH assay**	**FRAP assay**	**ORAC assay**	**BCB assay**
TPC	NA	0.951^a^	-0.575	-0.668	0.717	0.847^b^	-0.797
TFC	0.951^a^	NA	-0.553	-0.584	0.754	0.935^a^	-0.480
ABTS assay	-0.575	-0.553	NA	0.962^a^	-0.858^b^	-0.606	-0.108
DPPH assay	-0.668	-0.584	0.962^a^	NA	-0.856^b^	-0.590	0.272
FRAP assay	0.717	0.754	-0.858^b^	-0.856^b^	NA	-0.684	-0.096
ORAC assay	0.847^b^	0.935^a^	-0.606	-0.590	0.684	NA	-0.172
BCB assay	-0.797	-0.480	-0.108	0.272	-0.096	-0.172	NA

^a^Correlation is significant at the 0.01 level (2-tailed)

^b^Correlation is significant at the 0.05 level (2-tailed)

A sophisticated approach for analysing the interaction of herbs in TC-16 was done by calculating the CI and percentage difference in the antioxidant activity (Table 4).

**Table 4:** Interaction analysis in the different assays

Antioxidant capacity tests	CI values	Difference (%)
ABTS assay	4.688	-
DPPH assay	3.549	-
BCB assay	0.300	-
FRAP assay	-	-52.63
ORAC assay	-	96.37

CI value equal, smaller or bigger than 1 indicated addition, synergism and antagonism

Positive difference values (%) indicate potential synergistic and negative values antagonistic effects

## Discussion

### Determination of phenolic and flavonoid contents and antioxidant capacity tests

It has been widely known that plant natural products possess antioxidant activity. Among the phytochemicals present, phenolic compounds, with flavonoids being one of the major phenolics, play an important role in contributing to this activity by acting as an electron donor, hydrogen donor and by chelating metal ions [20]. TPC ranges from 1.42 ± 0.29 mg GAE/g (*A. dorsata* honey) to 66.08 ± 0.87 mg GAE/g (*C. longa*) whereas TFC ranges from 9.42 ± 0.77 mg CE/g (*C. microcarpa*) to 200.35 ± 1.47 mg CE/g (*C. longa*). Both TPC and TFC revealed that TC-16 possessed the most abundant phenolic and flavonoid contents (46.14 ± 1.40 mg GAE/g and 132.69 ± 1.43 CE/g respectively) following *C. longa.* This suggests why *C. longa* possessed numerous health benefits, inter alia, potent antioxidant and anti-microbial activities [21]. The high phenolic and flavonoid contents in TC-16 suggests the potential benefits of TC-16 to human health since these phytochemicals have long been associated with various biochemical and pharmacological properties [22].

The medicinal values of plant extracts are largely associated with their antioxidant property and ability to scavenge free radicals. Over the last few decades, numerous methods have been advanced to evaluate the antioxidant potential of various samples. However, performing a single assay is usually insufficient to fully understand the full antioxidant potential of samples due to differences in the reaction mechanisms, radicals produced, and assay parameters used. Hence, Schlesier et al. strongly advise that at least two different methods should be used [23]. For that reason, several in vitro antioxidant assays, namely ABTS, DPPH, FRAP, ORAC and BCB assays were employed in this study to evaluate the antioxidant potential of TC-16.

The in vitro antioxidant capacity assays can be classified into two types, single electron transfer (SET)-based assays and hydrogen atom transfer (HAT)-based assays. SET methods rely on the ability of the antioxidant to transfer an electron and reduce certain compounds and molecules while the basis of HAT methods lies on the ability of the antioxidant to scavenge reactive oxygen species (ROS) by donating a hydrogen ion from a stable molecule [24]. FRAP assay is based on SET mechanism, ORAC and BCB assays involve HAT whereas DPPH and ABTS utilize both HAT and SET [24, 25].

SET-based FRAP assay measures the ability of electron-donating antioxidants to reduce ferric iron (Fe^3+^) to ferrous ion (Fe^2+^). It is conducted at an acidic pH of 3.6 for iron solubility and the ionization potential is also reduced at low pH, driving the HAT mechanism [26]. The equation of the standard curve was y = 0.0109 x – 0.0087 (r^2^ = 0.9991). Consistent with ABTS and DPPH assays, among the extracts and polyherbal tested, the FRAP assay revealed that *C. longa* (12.92 ± 1.03 μM FeSO_4_/μg) exhibited the highest antioxidant activity, followed by *P. nigrum* (6.79 ± 0.95 μM FeSO_4_/μg) or *Z. officinale* (6.36 ± 0.29 μM FeSO_4_/μg) and TC-16 (12.92 ± 1.03 μM FeSO_4_/μg). These three assays demonstrated that no significant improvement in the antioxidant activity was yielded by the mixture of herbs in TC-16 as compared to the individual herb *C. longa, Z. officinale* and *P. nigrum*.

ORAC assay is a method that can be used to measure the total antioxidant capacity of biological fluids, food products and natural products [27–29]. This assay measures the hydrogen atom donating ability of antioxidants by monitoring the inhibition of peroxyl radical-induced oxidation, which is reflected as protection against quenching of fluorescent probe signal by antioxidants. Combining both the percentage and time of inhibition of free radical action by antioxidants in a single quantity, this assay has a distinct chemical meaning compared with the end-point results [30]. By plotting the net AUC against Trolox concentrations in the range of 6.25—100 μM, a calibration curve with the equation of y = 0.3785 x + 2.5946 with an excellent correlation coefficient (r^2^ = 0.9989) was obtained. Similarly, *C. longa* (613.04 ± 64.88 μmol TE/g) exhibited the highest antioxidant activity as reflected by the highest ORAC value compared with the polyherbal formulation and other ingredients. Despite, ORAC assessment of the samples gave a different antioxidant activity trend in which TC-16 exhibited the highest antioxidant activity after *C. longa* whereas, in other assays, *Z. officinale* and *P. nigrum* demonstrated higher antioxidant activity than TC-16. ORAC is considered to be biologically relevant as it deals with the peroxyl radical from biological systems [31]. The high ORAC value of TC-16 suggests that HAT is the dominant antioxidant mechanism of TC-16.

In the β-carotene/linoleic model, the oxidation of linoleic acid produces hydroperoxide-derived free radicals. Without the presence of an antioxidant, β-carotene reacts with free radicals, causing rapid bleaching of the yellow solution. Competition reaction takes place with the presence of another antioxidant and this can hinder the extent of β-carotene destruction, resulting in slower decolourisation of the solution [16]. The results obtained from the β-carotene bleaching assay also support HAT as the main antioxidant mechanism for TC-16, as demonstrated by its lowest value of IC_50_ (109.07 ± 4.43 μg/mL) after Trolox (IC_50_: 1.63 ± 0.22 μg/mL). Ascorbic acid, *C. microcarpa* and *A. dorsata* honey were incapable of inhibiting β-carotene bleaching by scavenging linoleate-derived free radicals, and this could be explained by the “polar paradox” theory [32]. The polarity of the extract may play a role in water: oil (w/o) emulsions in which apolar antioxidants are more effective antioxidants because they concentrate within the lipid phase, ensuring the protection of the emulsion itself. On the other hand, polar antioxidants such as ascorbic acid and those present in *C. microcarpa* and *A. dorsata* honey remain in the aqueous phase and will be markedly diluted, thus less effective in protecting the lipid [33, 34]. This suggests that the more potent antioxidant activity of TC-16 could be the result of apolar antioxidants present in TC-16.

ABTS assay studies the potential of antioxidants in scavenging the blue-green radical ABTS^•+^ cation at 734 nm into the colourless ABTS form, where the intensity will be reduced with the presence of antioxidants. Based on the results obtained, *C. longa* (IC_50_: 12.74 ± 0.27 μg/mL) and *Z. officinale* (IC_50_: 28.49 ± 0.73 μg/mL) showed the highest ABTS radical scavenging activity among the herbs after the positive controls ascorbic acid (IC_50_: 2.07 ± 0.31 μg/mL) and Trolox (IC_50_: 5.41 ± 0.61 μg/mL). The polyherbal TC-16 showed moderate antioxidant activity (IC_50_: 151.10 ± 0.95 μg/mL). DPPH assay measures the ability of antioxidants to reduce the purple DPPH radical into the yellow α, α-diphenyl-β-picrylhydrazine. In this assay, TC-16 also showed a moderate antioxidant activity with IC_50_ of 170.83 ± 8.64 μg/mL. Most of the samples demonstrated a lower IC_50_ value in ABTS assay as compared with DPPH assay as DPPH assay only takes into account the hydrophobic antioxidants whereas ABTS assay considers both hydrophilic and lipophilic antioxidants [35]. This suggests that, in addition to hydrophobic antioxidants, hydrophilic antioxidants also played a role in their antioxidant property. On the contrary, *Z. officinale* showed greater activity in the DPPH assay which is consistent with several studies [36, 37].

### Correlation and interaction analysis

The positive correlation between TPC and TFC might be due to flavonoids being one of the major phenolic compounds in these extracts. Increased TPC and TFC may be associated with increased antioxidant activities, which was indicated by lower IC_50_ values for ABTS, DPPH and BCB assays, and higher FRAP and ORAC values. Hence, TPC and TFC were negatively correlated with the IC_50_ of DPPH, ABTS and BCB assays and positively correlated with FRAP and ORAC values, consistent with the results reported by Al-Laith et al. [38] and Ruslan et al. [39]. However, a significant correlation was only found between the ORAC assay with TPC and TFC, indicating that phenolic and flavonoid contents were the major contributors to antioxidant activity in the ORAC assay, while other phytochemicals might have contributed to the antioxidant activity in other assays. Significant correlations were found between ABTS, DPPH and FRAP assays, especially between ABTS and DPPH (R = 0.962, p < 0.01), suggesting that these three methods have a similar predictive capacity for the antioxidant activities of TC-16. The lowest correlation was identified between the BCB assay and the others (R = -0.108, R = 0.272, R = -0.096 and R = -0.172 with ABTS, DPPH, FRAP and ORAC respectively). This may be attributed to the different antioxidant mechanisms, as ABTS, DPPH and FRAP assays rely mainly on the SET mechanism while ORAC and BCB are HAT-based assays, but ORAC also incorporates the kinetic action of antioxidants.

For the interaction analysis, the difference in antioxidant activity was calculated for FRAP and ORAC assays, with the positive values indicating synergism while negative values represent antagonism. Based on that postulate, the combination of herbs in TC-16 resulted in 96.37% better activity in the ORAC assay as compared to the results obtained from the individual herbs. However, the FRAP assay revealed that the combination of herbs reduced the antioxidant activity by 52.63%. To determine the interaction of herbs in TC-16 via DPPH, ABTS and BCB assays, CI values were calculated. CI values equal, smaller or greater than 1 suggest an addition, synergism or antagonism among herbs respectively. The calculated CI value indicates that antagonism occurred among the herbs as determined by their CI value in ABTS (4.688) and DPPH (3.549) assays, while the CI value in the BCB assay (0.300) reveals synergism. This was aligned with the findings in several studies showing that a combination of herbs does not always result in a synergistic antioxidant activity instead, antagonism might occur [40, 41]. This may be attributed to the presence of different kinds of flavonoids in the respective herbs, which promotes an antagonistic effect when combined [18].

## Conclusions

Alkaloids, flavonoids, terpenoids, saponins and glycosides were the phytochemicals present in TC-16 and TC-16, together with its individual ingredients possessed free radical scavenging and antioxidant activities in different assays. The antioxidant mechanism of TC-16 was mainly HAT-based, which may be attributed by the phenolics, and flavonoids present in it. The combination of herbs in TC-16 resulted in a decreased effect in all the antioxidant assays except for ORAC and BCB. This shows that synergistic interaction among the herbs in a PHF occur through a specific mechanism, which in this study, synergism in TC-16 occurs mainly via HAT-based mechanism.

Although we have demonstrated that interactions among the individual herbs may affect the total antioxidant capacity of the mixture, more detailed studies comprising the different combinations of herbs and proportions in the mixtures are crucial to gain a better comprehension of the mechanisms involved in the interactions. In addition, the identification of active compounds and in vivo safety must be thoroughly investigated prior to their possible applications. Mechanisms showing positive interactions should be focused in order to maximise the benefits of a PHF.

## Data Availability

The data used to support the findings of this study are available from the first or corresponding authors upon request.

## Abbreviations

ABTS: 2,2’-Azino-bis(3-ethylbenzothiazoline-6-sulfonate)
AUC: Area under the curve
BCB: β-Carotene bleaching
BHA: Butylhydroxyanisole
BHT: Butylhydroxytoluene
CE: Catechin equivalent
CI: Combination index
DPPH: 2,2-Diphenyl-1-picrylhydrazyl
FRAP: Ferric reducing antioxidant power
GAE: Gallic acid equivalent
HAT: Hydrogen atom transfer
ORAC: Oxygen radical absorbance capacity
PHF: Polyherbal formulation
SET: Single electron transfer
TE: Trolox equivalent
TFC: Total flavonoid content
TPC: Total phenolic content
TPTZ: 2,4,6-Tris(2-pyridyl)-s-triazine
w/o: Water: oil
